# Supplementary material for: Decoding locomotion from population neural activity in moving C. elegans
Source: eLife. 2021 Jul 29;10:e66135. doi: 10.7554/eLife.66135 (PMC8439659; doi:10.7554/eLife.66135)
Supplement: Transparent reporting form [file elife-66135-transrepform.docx]

***eLife’s* transparent reporting form**

We encourage authors to provide detailed information *within their submission* to facilitate the interpretation and replication of experiments. Authors can upload supporting documentation to indicate the use of appropriate reporting guidelines for health-related research (see [EQUATOR Network](http://www.equator-network.org/%20)), life science research (see the [BioSharing Information Resource](https://biosharing.org/)), or the [ARRIVE guidelines](http://www.plosbiology.org/article/info:doi/10.1371/journal.pbio.1000412) for reporting work involving animal research. Where applicable, authors should refer to any relevant reporting standards documents in this form.

If you have any questions, please consult our Journal Policies and/or contact us: [editorial@elifesciences.org](mailto:editorial@elifesciences.org).

**Sample-size estimation**

- You should state whether an appropriate sample size was computed when the study was being designed
- You should state the statistical method of sample size computation and any required assumptions
- If no explicit power analysis was used, you should describe how you decided what sample (replicate) size (number) to use

Please outline where this information can be found within the submission (e.g., sections or figure legends), or explain why this information doesn’t apply to your submission:

No explicit power analysis was used. We sought to match or exceed the number of recordings used in comparable published work.

**Replicates**

- You should report how often each experiment was performed
- You should include a definition of biological versus technical replication
- The data obtained should be provided and sufficient information should be provided to indicate the number of independent biological and/or technical replicates
- If you encountered any outliers, you should describe how these were handled
- Criteria for exclusion/inclusion of data should be clearly stated
- High-throughput sequence data should be uploaded before submission, with a private link for reviewers provided (these are available from both GEO and ArrayExpress)

Please outline where this information can be found within the submission (e.g., sections or figure legends), or explain why this information doesn’t apply to your submission:

Criteria for inclusion of a recording is described explicitly in the Methods section “Whole brain imaging” in the subsections titled “Whole brain imaging in moving animals” and “Moving to immobile transition experiments.” All recordings used in this work have been made publicly accessible online with a permanent DOI (10.17605/OSF.IO/DPR3H). Table 3 in the manuscript lists all recordings used in this study. Table 4 further specifies exactly which recordings are included in which figure subpanels. Figure captions also list the number of recording (or, when relevant the number of neurons). Box plots used in the manuscript all show individual data points.

**Statistical reporting**

- Statistical analysis methods should be described and justified
- Raw data should be presented in figures whenever informative to do so (typically when N per group is less than 10)
- For each experiment, you should identify the statistical tests used, exact values of N, definitions of center, methods of multiple test correction, and dispersion and precision measures (e.g., mean, median, SD, SEM, confidence intervals; and, for the major substantive results, a measure of effect size (e.g., Pearson's r, Cohen's d)
- Report exact p-values wherever possible alongside the summary statistics and 95% confidence intervals. These should be reported for all key questions and not only when the p-value is less than 0.05.

Please outline where this information can be found within the submission (e.g., sections or figure legends), or explain why this information doesn’t apply to your submission:

Statistical analysis methods for the analyses used in this work are described in the Methods in the section “Relating neural activity to behavior.” In particular, significance testing of tuning curves is described in the “Tuning Curves” subsection. Details of the decoding models are described in the subsections “Population Model,” “Alternative models,” and “Restricted Models.” Statistical analysis of dissimilarity in correlation matrices is described in the methods section “Correlation structure analysis.”

P-values and the type of significance tests are reported in figure legends and also in the main text when each relevant result is discussed. For tuning curves shown in Figure 1 – Figure Supplement 2 and 3, p-values are shown in the figure itself.

(For large datasets, or papers with a very large number of statistical tests, you may upload a single table file with tests, Ns, etc., with reference to sections in the manuscript.)

**Group allocation**

- Indicate how samples were allocated into experimental groups (in the case of clinical studies, please specify allocation to treatment method); if randomization was used, please also state if restricted randomization was applied
- Indicate if masking was used during group allocation, data collection and/or data analysis

Please outline where this information can be found within the submission (e.g., sections or figure legends), or explain why this information doesn’t apply to your submission:

No randomized groupings were used.

**Additional data files (“source data”)**

- We encourage you to upload relevant additional data files, such as numerical data that are represented as a graph in a figure, or as a summary table
- Where provided, these should be in the most useful format, and they can be uploaded as “Source data” files linked to a main figure or table
- Include model definition files including the full list of parameters used
- Include code used for data analysis (e.g., R, MatLab)
- Avoid stating that data files are “available upon request”

Please indicate the figures or tables for which source data files have been provided:

Code for data analysis is available at: <https://github.com/leiferlab/PredictionCode/> All data used in this study is posted in public Open Science Foundation repository with a permanent DOI, 10.17605/OSF.IO/DPR3H.
